# The construct validity and responsiveness of the EQ-5D, SF-6D and Diabetes Health Profile-18 in type 2 diabetes

**DOI:** 10.1186/1477-7525-12-42

**Published:** 2014-03-24

**Authors:** Brendan Mulhern, Keith Meadows

**Affiliations:** 1 Health Economics and Decision Science, School of Health and Related Research, University of Sheffield, Regent Court S1 4DA, UK; 2 DHP Research and Consultancy, Bloxham Mill Business Centre, Barford Road, Bloxham, Banbury OX15 4FF, UK

**Keywords:** EQ-5D, SF-6D, DHP, Psychometrics, Validity

## Abstract

**Background:**

Interest in the measurement of health related quality of life and psychosocial functioning from the patient’s perspective in diabetes mellitus has grown in recent years. The aim of this study is to investigate the psychometric performance of and agreement between the generic EQ-5D and SF-6D and diabetes specific DHP-18 in Type 2 diabetes. This will support the future use of the measures by providing further evidence regarding their psychometric properties and the conceptual overlap between the instruments. The results will inform whether the measures can be used with confidence alongside each other to provide a more holistic profile of people with Type 2 diabetes.

**Methods:**

A large longitudinal dataset (n = 1,184) of people with Type 2 diabetes was used for the analysis. Convergent validity was tested by examining correlations between the measures. Known group validity was tested across a range of clinical and diabetes severity indicators using ANOVA and effect size statistics. Agreement was examined using Bland-Altman plots. Responsiveness was tested by examining floor and ceiling effects and standardised response means.

**Results:**

Correlations between the measures indicates that there is overlap in the constructs assessed (with correlations between 0.1 and 0.7 reported), but there is some level of divergence between the generic and condition specific instruments. Known group validity was generally good but was not consistent across all indicators included (with effect sizes from 0 to 0.74 reported). The EQ-5D and SF-6D displayed a high level of agreement, but there was some disagreement between the generic measures and the DHP-18 dimensions across the severity range. Responsiveness was higher in those who self-reported change in health (SRMs between 0.06 and 0.25).

**Conclusions:**

The psychometric assessment of the relationship between the EQ-5D, SF-6D and DHP-18 shows that all have a level of validity for use in Type 2 diabetes. This suggests that the measures can be used alongside each other to provide a more holistic assessment of with the quality of life impacts of Type 2 diabetes.

## Introduction

Interest in the measurement of health related quality of life (HRQL) and psychosocial functioning from the patient’s perspective in diabetes mellitus has grown in recent years. Diabetes is a chronic disease with a range of related health complications including heart disease, stroke, and kidney, feet and eye complications. Type 2 diabetes generally occurs in later life and is caused when the body does not produce enough insulin. Health concerns related to Type 2 diabetes impact on an individual’s level of HRQL, including mental health [1,2] and social activities [3]. Assessing HRQL in diabetes alongside related clinical factors therefore allows the impact of the condition and different treatments on areas of health and functioning that are important to the person with diabetes to be measured. Both generic and diabetes specific patient reported outcome measures (PROMS) can be used, and administering generic and condition specific measures together can provide a more detailed profile of the HRQL impacts of diabetes. However to ensure valid and reliable measurement, it is important to investigate the psychometric performance of both generic and condition specific measures of health status in diabetes, and also explore the relationship between instruments.

Generic preference based measures (GPBMs) such as EQ-5D [4,5] and SF-6D [6,7] can be used in diabetes to measure health status and HRQL. GPBMs can also be used in the economic evaluation of interventions using the Quality Adjusted Life Year (QALY) as the outcome measure. QALYs combine values for quality and length of life into a single figure. GPBMs are scored using a utility scale (that is the quality weight of the QALY) which is derived by asking the general population to provide preferences for health states defined by the descriptive system of the measure. This is used to model utility value for every health state anchored on a 1 (full health) to 0 (dead) scale (where negative values are equivalent to states valued as worse than dead). A condition specific PROM that can be used to assess the relationship between Type 2 diabetes and psychosocial functioning is the Diabetes Health Profile [8]. The DHP-18 has been adopted by the Department of Health for their Long Term Conditions Patient Reported Outcome Measures PROMs pilot [9].

The generic nature of the EQ-5D and SF-6D means that it is important to assess the conditions in which the measures perform well. There is some evidence to suggest that the EQ-5D is valid for use in Type 2 diabetes. In a recent review, Janssen and colleagues [10] found evidence for the construct validity and responsiveness to change of the EQ-5D, but there was also the suggestion of the ceiling effect. Kontodimopoulos and colleagues [11] found that the EQ-5D and SF-6D were sensitive to a number of diabetes related complications. Meadows et al. [8] found evidence for the construct validity and patient acceptability of the DHP-18 for use in Type 2 diabetes, but further evidence regarding responsiveness to change in health status is required.

Although some evidence exists about the psychometric properties of the measures, it is important to assess this across multiple samples, and further work is required to provide a wider range of evidence for the measures, particularly in relation to each other. Therefore the aim of this study is to investigate the construct validity and responsiveness of the EQ-5D, SF-6D and DHP-18 in a large sample of people with Type 2 diabetes, and also to compare the measures. This will support the future use of the measures by providing further evidence regarding their psychometric performance. The future use of these measures is desirable, as the DHP-18 has been tested as part of the UK Department of Health’s Long Term Conditions PROMs pilot, and there is interest in using PROMs in long term conditions to track patient change (both clinically and for the individual), and assess service performance. There is also an ongoing need to use GPBMs for the economic evaluation of interventions, and use of the EQ-5D in particular is widespread in population and health surveys. The results will also help to establish whether the measures are valid, and can therefore be used with confidence alongside each other to provide complementary information that allows for a more detailed picture of the HRQL of people with Type 2 diabetes to be gained. This in turn can inform clinical decision making. The results regarding the EQ-5D and SF-6D can also potentially improve confidence in the values used for economic evaluations carried out for diabetes specific interventions.

## Methods

### Measures

#### EQ-5D

The EQ-5D [4,5] is a widely used generic preference based measure that assesses health status across five dimensions (mobility, self care, usual activities, pain/discomfort, anxiety/depression) with three response levels (therefore generating 243 (3^5^) health states in total). The utility scale for use in economic evaluation was derived using the preference elicitation technique Time Trade Off and ranges from −0.584 to 1.00. A Visual Analogue Scale (VAS) can also be part of the EQ-5D system, but is not included in this analysis. The EQ-5D is the measure recommended for use in the cost utility analysis of new interventions and treatments by the UK National Institute for Health and Care Excellence [12].

#### SF-6D

The SF-6D [6,7] is a generic preference based measure derived from SF-36/SF-12 that assesses health on six dimensions (physical functioning, role functioning, social functioning, pain, mental health, vitality) with four to six response options (thereby describing 18,000 health states). It generates a preference based utility scale (range 0.29 to 1) that was derived using the preference elicitation technique Standard Gamble. SF-6D is accepted by reimbursement agencies in Australia [13] and Canada [14].

#### DHP-18

The DHP-18 [8] was developed from the DHP-1 [15] and assesses psychosocial functioning in Type 2 diabetes across three dimensions: Psychological Distress (PD; 6 items); Barriers to Activity (BA; 7 items); Disinhibited Eating (DE; 5 items). Each of the 18 items is scored on a 0 to 3 scale (never, sometimes, usually, always), and dimensions rescored on a 0–100 scale by dividing the raw score for each dimension by the overall score range, and multiplying this by 100. High scores are indicative of lower levels of health. The DHP-18 is used in a range of settings including clinical trials and population health surveys [16-19]. It has been translated to 29 languages and can be completed using a range of media including face to face, paper and pencil, and internet or mobile versions.

### Sample

The sample was taken from a longitudinal dataset (baseline and 1 year follow up) from a UK community-based postal survey of people with Type 2 diabetes in one local health board area [18]. The aim of the study was to investigate the HRQL of the population with diabetes following service restructuring. Respondents were identified from primary care diabetes patient registers, and 13 of 19 General Practitioner practices in the area agreed to take part in the study. Those over 18 who were not pregnant were included. In total, 4,040 people with both Type 1 and Type 2 diabetes were approached to take part in the study, with 1,613 (40%) people with Type 2 diabetes responding at baseline and 1,184 (29%) responding at follow up. In this study, the 1,184 people who responded at both time points were included to allow for the same sample to be used across all analyses. Table 1 provides the demographic characteristics of this sample.. It was found that no specific group of diabetics were prone to non-response at follow up [18]. Missing data rates were low at baseline (1%-2% for all measures) and follow up (3%-5%). Missing data was not imputed for the EQ-5D, SF-6D or DHP-18 as missing data rates were low. Furthermore t is recommended that missing DHP-18 data is not imputed when testing the psychometrics of the measure [20].

**Table 1:** Background characteristics

**Demographic category**		**N (%)**
*N*		1184
*Age (m,sd)*		66.6 (10.8)
*Age*		
18-45		45 (3.8)
46-60		281 (23.7)
61-70		394 (33.3)
71-80		346 (29.3)
81+		114 (9.7)
*Gender*		
Male		720 (60.9)
*Marital status*		
Single		118 (10.0)
Married/partner		891 (75.2)
Divorced		74 (6.3)
Widowed		99 (8.4)
*Length of time with diabetes*		
< 5 years		612 (51.7)
> = 5 years		572 (48.3)
*Diabetes management*		
Diet		321 (27.1)
Tablets		711 (60.1)
Insulin		148 (12.5)
*Health status*		
*Diabetes related complications*	None	710 (61.3)
One or more		448 (38.7)
Feet		257 (21.7)
Eyes		266 (22.5)
Kidneys		66 (5.6)
*Other health complications*	None	216 (18.5)
One or more		952 (81.5)
Arthritis		503 (43.2)
Hypertension		513 (44.1)
High cholesterol		588 (50.5)
Heart conditions		108 (11.4)
Depression/anxiety		207 (17.8)
*EQ-5D index score*		0.652 (0.32)
*SF-6D index score*		0.693 (0.16)
*DHP-18* PD		17.93 (20.5)
BA		21.53 (19.31)
DE		35.93 (23.04)
*Change in health baseline-follow up*		
Health improvement		186 (15.9)
No change		717 (61.7)
Health deterioration		260 (22.4)

The majority of the sample (60%) were male, and aged over 60 years old (72%). The length of time respondents has been diagnosed with diabetes ranged from 1 week to 51 years, with 52% being diagnosed less than 5 years. Diabetes management regimes included diet only (27%), tablets (60%) and insulin (12.5%). At baseline, 38.7% reported diabetes related health complications (including complications of the feet, eyes and kidneys) and 80.4% reported other health complications (including arthritis, hypertension, high cholesterol heart conditions and depression/anxiety). Between baseline and follow up, the majority of the sample (62%) reported no change in general health status, with 16% reporting improvement and 22% reporting deterioration.

### Psychometric analysis

A range of psychometric tests were carried out to assess the construct validity and responsiveness of the measures in relation to each other and across different clinical and severity indicators, and these are described below.

#### Construct validity

Construct validity assesses how well an instrument measures what it was intended to measure. Two types of construct validity, defined as convergent and known group validity, were assessed. It should be noted that there is no gold standard for the measurement of outcomes in diabetes, and this is due to the heterogeneous impacts of the condition on HRQL and psychosocial functioning. Therefore the psychometric validity of instruments in relation to each other and to external clinical indicators can only be implied rather than proved.

#### Convergent validity

Convergent validity assesses the relationship between measures in terms of whether they are measuring overlapping constructs (in this case health status and HRQL as measured by the generic measures and psychological and behavioural functioning as measured by the DHP-18). The convergence between the GPBM utility and dimension scores and the rescaled DHP-18 dimension scores was assessed using Pearson’s correlation coefficients. High correlations (where correlations ≥0.7 are strong, <0.7 to ≥0.3 are moderate and <0.3 are weak) indicates that the measures are assessing similar diabetes-related constructs.

#### Known group validity

Known group validity assesses the extent to which the EQ-5D, SF-6D and DHP-18 dimensions discriminate between different clinical and/or severity groups as defined by other indicators. In this study known group validity was assessed in comparison to groups defined by the presence or absence of diabetes related and other comorbidities at the overall level (i.e. one group reporting problems and one group reporting no problems) and also for specific conditions (diabetes problems were feet, eye and kidney related, and other comorbidities included arthritis, hypertension, high cholesterol, heart disease and anxiety/depression). We also used the length of time with diabetes (as it is a chronic condition where the HRQL impacts worsen over time, and there is some evidence for differences in PROM scores using this category [10]); and diabetes treatment regime (as the treatment or advised method of control for Type 2 diabetes may also be a proxy for severity) as indicators to assess known group validity. In this case groups were defined as diet only (least severe), tablets, and insulin (most severe). Mean scores on the measures, one way ANOVA significance tests and effect sizes (calculated by dividing the difference between the mean values for each group by the standard deviation of the milder severity group) were used to assess the magnitude and direction of the differences across the severity groups. Effect sizes of less than 0.2 are considered small, 0.5 moderate, and 0.8 large [21].

#### Agreement between EQ-5D, SF-6D and DHP-18 dimension scores

Agreement between the measures was investigated using Bland Altman plots [22]. These charts plot the mean of a pair of scores on the x axis, and the difference between the pair of scores on the y axis. This allows agreement in terms of the difference between the scores to be assessed across the full severity range, which is done by adding upper and lower boundaries plus or minus two standard deviations away from the mean difference in score on the y axis. Outliers are defined as points outside of these boundaries. To allow for an assessment of the relationship between the utility measures and the the DHP-18, the dimension scores were rescored on a 0 to 1 scale, and reversed so that a low score was indicative of increased impairment in line with the GPBMs. This allows for comparison on the same scale which is required for bland altman plots, and was done for the agreement analysis only.

#### Responsiveness

Responsiveness assesses the sensitivity of PROMs to change in health status over time and is an important measurement property. Responsiveness was assessed using the standardised response mean statistic (calculated by dividing the mean change on the measure by the standard deviation of the change). SRM categories as defined as follows: small: >0.2 ≤ 0.5, moderate: >0.5 < 0.8, large: ≥0.8 [22]. Responsiveness was tested for the overall sample, and also by groups self-reporting improvement, deterioration or no change in their health status. Floor (% at the lowest level of dysfunctioning) and ceiling effect (% at the highest degree of dysfunctioning) tests were also carried out. If a large proportion of the sample is at the floor or ceiling, the ability of the measure to detect any deterioration or improvement in health status is impaired.

## Results

### Convergent validity

Correlations between the measures are shown in Table 2. Negative correlations appear in the table as high score on an EQ-5D or SF-6D dimension is indicative of poor health status, but a high score on the utility scale is indicative of better health (i.e. on the full health (1) to dead (0) utility scale). The same is the case for the DHP-18 dimension scores and the utility scores, where a high score on the DHP is indicative of lower psychosocial functioning. Evidence of moderate to strong convergence between the EQ-5D and SF-6D index and dimension scores was identified, indicating that both generic measures are assessing similar constructs. Moderate convergence between the EQ-5D, SF-6D and DHP dimensions was identified, with the PD dimension correlating highest with the SF-6D mental health dimension and BA dimension correlating highest with the SF-6D index, role and social scales. This demonstrates overlap between the constructs being measured on the GPBMS and the PD and BA dimensions but a range of low correlations as the GPBM dimension level indicates that the measures are covering a range of divergent HRQL factors. However convergence between the GPBMs and the disinhibited eating factor was on the low side indicating a lower level of overlap between the constructs.

**Table 2:** Convergence between the measures

	**SF-6D**							**DHP**		
	**Index**	**Physical**	**Role**	**Social**	**Pain**	**Mental**	**Vitality**	**PD**	**BA**	**DE**
EQ-5D										
Index score	0.76	−0.63	−0.55	−0.68	−0.73	−0.45	−0.55	−0.41	−0.43	−0.21
Mobility	−0.62	0.63	0.51	0.50	0.62	0.24	0.50	0.21	0.30	0.10
Self care	−0.53	0.54	0.40	0.50	0.50	0.24	0.40	0.26	0.35	0.12
Usual activities	−0.70	0.67	0.55	0.63	0.63	0.35	0.55	0.30	0.36	0.14
Pain/discomfort	−0.65	0.54	0.46	0.53	0.73	0.33	0.49	0.32	0.34	0.17
Anxiety/depression	−0.57	0.29	0.53	0.50	0.41	0.65	0.37	0.58	0.443	0.32
DHP										
PD	−0.46	0.23	0.40	0.41	0.32	0.52	0.32	-	-	-
BA	−0.49	0.34	0.40	0.47	0.37	0.39	0.36	-	-	-
DE	−0.25	0.06	0.22	0.11	0.17	0.28	0.23	-	-	-

All correlations significant at 0.01 level.

### Known group validity

Table 3 displays the mean scores for the measures across a range of different clinical and severity groups (with significant P values in italics). One way ANOVA demonstrated that the EQ-5D, SF-6D and DHP-18 dimensions significantly discriminated between patients with and without diabetes related problems (all *p* < 0.01), with effect sizes in the moderate range for all but the DE dimension. For specific problems, all of the measures apart from the DHP-18 DE dimension significantly differed across those with and without foot problems (*p* < 0.01), but effect sizes were small. For eye problems, the SF-6D (*p* = 0.02) and BA dimension (*p* < 0.01) were sensitive to differences with a small effect size. None of the measures were significantly different across samples with and without kidney problems, but this is linked to the small amount of people reporting an issue.

**Table 3:** Known group validity of the EQ-5D, SF-6D and DHP-18

**Category**^**1**^		**EQ-5D**			**SF-6D**			**DHP-18 (PD)**			**DHP-18 (BA)**			**DHP-18 (DE)**		
		**M**	**P**	**ES**	**M**	**P**	**ES**	**M**	**P**	**ES**	**M**	**P**	**ES**	**M**	**P**	**ES**
Diabetes related health problems																
Overall	Yes	0.54	*<0.01*	0.74	0.63	*<0.01*	0.66	24.20	*<0.01*	0.62	28.57	*<0.01*	0.73	38.11	*<0.01*	0.16
	No	0.73			0.73			13.70			16.80			34.46		
Foot related	Yes	0.49	<*0.01*	0.38	0.62	*0.01*	0.20	26.59	*0.02*	0.25	30.36	*0.04*	0.20	39.14	0.32	0.09
	No	0.61			0.65			21.15			26.14			37.00		
Eye related	Yes	0.52	0.12	0.16	0.62	*0.02*	0.20	25.87	0.09	0.17	30.76	*0.01*	0.28	39.49	0.15	0.15
	No	0.57			0.65			21.97			25.26			36.35		
Kidney related	Yes	0.57	0.35	0.11	0.65	0.36	0.13	19.90	0.10	0.26	26.23	0.34	0.13	36.10	0.41	0.11
	No	0.53			0.63			25.07			28.97			38.60		
Comorbid health problems																
Overall	Yes	0.61	*<0.01*	1.10	0.67	*<0.01*	1.17	19.73	*<0.01*	0.70	22.92	*<0.01*	0.55	37.66	*<0.01*	0.43
	No	0.84			0.81			9.86			14.54			28.27		
Arthritis	Yes	0.48	*<0.01*	1.08	0.61	*<0.01*	1.08	22.00	*<0.01*	0.24	26.39	*<0.01*	0.42	38.90	0.08	0.11
	No	0.76			0.73			17.12			19.00			36.31		
Hypertension	Yes	0.62	0.64	0.03	0.67	0.52	0.07	19.69	0.96	0.00	23.37	0.46	0.05	38.13	0.52	0.04
	No	0.61			0.66			19.75			22.42			37.16		
High cholesterol	Yes	0.61	0.90	0.00	0.67	0.35	0.06	19.69	0.96	0.00	23.33	0.43	0.00	38.12	0.45	0.05
	No	0.61			0.66			19.76			22.28			36.97		
Heart disease	Yes	0.48	*<0.01*	0.47	0.61	*<0.01*	0.40	18.76	0.63	0.05	25.34	0.18	0.14	32.32	*0.01*	0.26
	No	0.63			0.67			19.84			22.63			38.37		
Anxiety/depression	Yes	0.45	*<0.01*	0.70	0.57	*<0.01*	0.75	34.12	*<0.01*	1.02	31.30	*<0.01*	0.59	46.72	*<0.01*	0.53
	No	0.66			0.69			15.69			20.57			35.14		
Length of time diagnosed	< 5 years	0.66	0.41	0.06	0.70	0.08	0.13	17.18	0.18	0.08	18.52	*<0.01*	0.35	35.92	0.91	0.01
	> = 5 years	0.64			0.68			18.82			24.77			35.76		
Treatment regime	Diet	0.69	*<0.01*	0.10	0.71	*<0.01*	0.06	12.76	*<0.01*	0.36	14.49	*<0.01*	0.52	32.75	*0.01*	0.17
	Tablets	0.66		0.42	0.70		0.44	18.62		0.29	22.04		0.65	36.81		0.08
	Insulin	0.53			0.63			24.58			34.49			38.59		

^1^The sample sizes for each category are included in Table 1.

All of the measures also differ across groups with and without comorbid problems not related to diabetes (all *p* < 0.01), with the GPBMs demonstrating large and the DHP-18 moderate effect sizes. When considering individual conditions, the level of differences is more mixed. Both the GPBMs and the DHP display differences across between groups defined by the presence or absence of arthritis and depression/anxiety (all *p* < 0.01), and the GPBMS and the DE dimension display significant differences across groups defined by presence or absence of heart disease (*p* < 0.01).

Assessment of scores across groups defined by length of time with diabetes shows that only the DHP-18 BA dimension demonstrates significant differences. Both the GPBMs and the DHP-18 significantly discriminate based on diabetes treatment regime, but effect sizes differ both across the measures and between the treatment categories.

### Agreement between the measures

**Figure 1:**
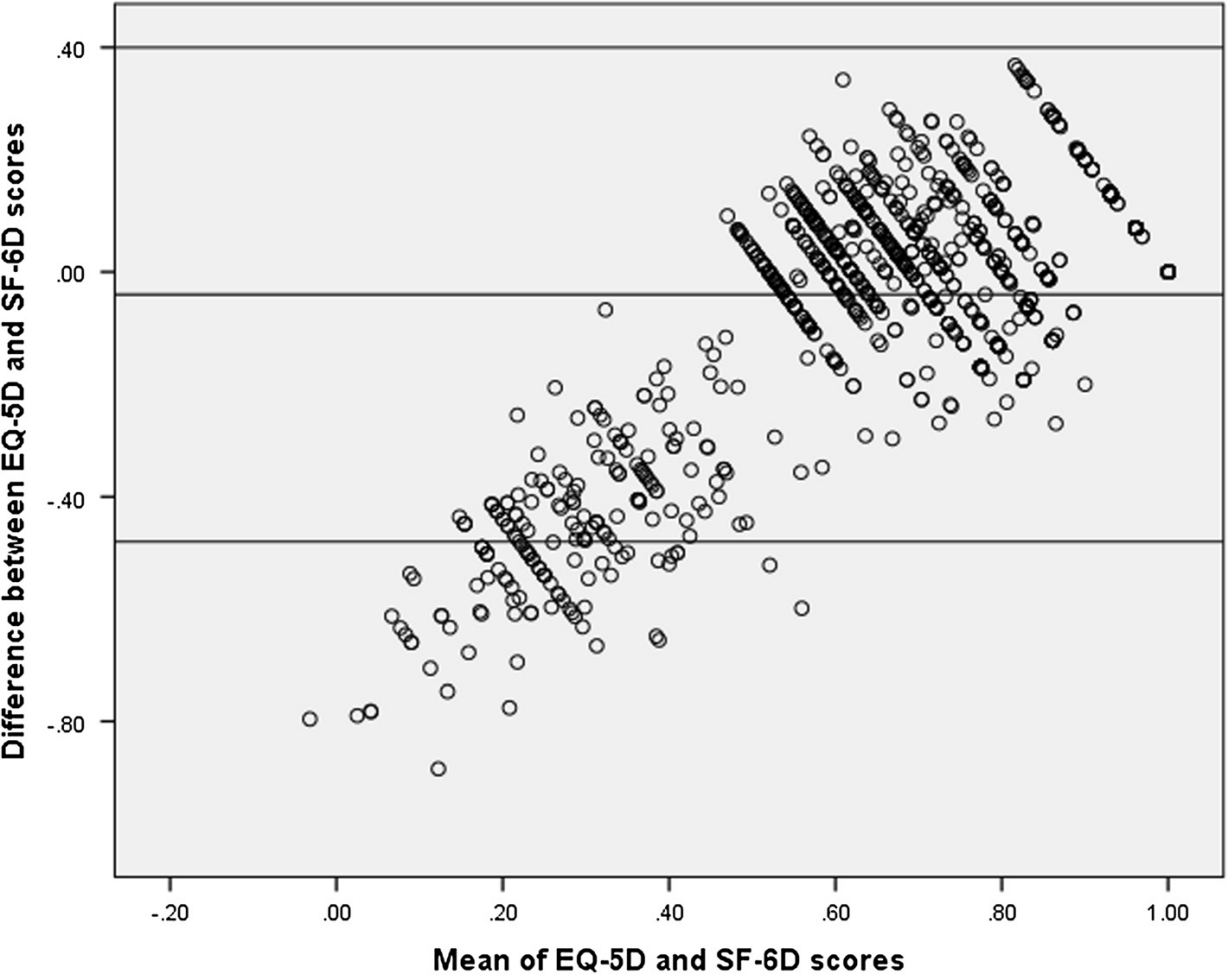
Agreement between EQ-5D and SF-6D.

**Figure 2:**
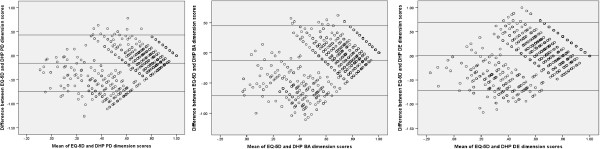
Agreement between EQ-5D and DHP-18 dimension scores.

**Figure 3:**
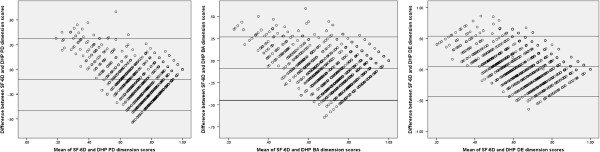
Agreement between SF-6D and DHP-18 dimension scores.

The Bland Altman plot of EQ-5D and SF-6D (Figure 1) indicates that agreement was lower where higher levels of HRQL impairment is reported (outside the lower boundary), but better at the milder end scale (where the majority of the values, which are all within the upper boundary, are found). Agreement between the GPBMS and the DHP-18 dimension scores is more mixed, with a lower level of agreement outside across the overall scale of impairment as measured by the instruments (Figures 2 and 3).

### Responsiveness

EQ-5D and the DHP-18 PD dimension have evidence of ceiling effects at both baseline and follow up. There was however, no evidence of a floor or ceiling effect for the SF-6D or DHP-18 BA and DE dimensions (Table 4). Overall, responsiveness of the instruments to change in reported outcome over time was in the range defined as small. For the health change subgroups it was found that responsiveness was higher for those self-reporting health improvement or deterioration which is as would be expected. However the SRMs were still small.

**Table 4:** Responsiveness by self-reported change in health status

	**% at floor**		**% at ceiling**		**Mean (sd) change**	**SRM**
	**T0**	**T1**	**T0**	**T1**		
EQ-5D						
Overall	0	0	23.3	23.2	−0.01 (0.23)	−0.04
Health improvement	0	0	27.6	31.0	0.04 (0.22)	0.18
No change	0	0	28.7	28.6	−0.01 (0.20)	−0.05
Health deterioration	0	0	6.2	2.8	−0.07 (0.29)	−0.24
SF-6D						
Overall	0	0	2.9	1.8	−0.01 (0.11)	−0.09
Health improvement	0	0	4.3	2.8	0.03 (0.12)	0.25
No change	0	0	3.5	2.3	−0.01 (0.10)	−0.10
Health deterioration	0	0	0.4	1.2	−0.03 (0.11)	−0.27
DHP-18 (PD)						
Overall	0.2	0.4	29.0	28.5	−0.28 (14.66)	−0.02
Health improvement	0	0	27.0	26.4	−1.91 (14.87)	−0.13
No change	0	0.3	33.3	32.5	−0.36 (13.62)	−0.03
Health deterioration	0.8	1.2	19.3	18.0	0.86 (16.08)	0.05
DHP-18 (BA)						
Overall	0.3	0.2	13.7	13.4	0.52 (13.95)	0.04
Health improvement	0	0	15.2	15.4	−2.74 (13.96)	−0.20
No change	0	0.1	15.7	15.1	0.46 (12.59)	0.04
Health deterioration	0	0.4	7.4	7.6	2.83 (17.01)	0.17
DHP-18 (DE)						
Overall	1.3	1.0	5.3	6.2	−0.48 (16.25)	−0.03
Health improvement	0.5	0	5.5	6.6	−4.25 (18.57)	−0.23
No change	1.3	0.7	5.2	6.4	−0.10 (15.33)	−0.01
Health deterioration	1.9	2.8	5.4	4.4	1.08 (16.71)	0.06

## Discussion

This study reports on the psychometric assessment of the relationship between the genericEQ-5D and SF-6D and the condition specific DHP-18 for use with Type 2 diabetes. The study provided supporting evidence for the construct validity of all three measures, as we found that the measures discriminate between groups with differing levels of health problems and diabetes specific issues. This is in line with previous findings regarding their psychometric properties in diabetes samples [8,10,11]. However the results need to be interpreted with caution due to the indicators used, where the GPBMs may be sensitive to the co-morbid problems being reported rather than diabetes-related HRQL factors per se. It is also interesting to note that the DHP-18 discriminates between groups defined by presence or absence of non-diabetes specific co-morbid conditions. This could be linked to the progressive nature of diabetes, where co morbid health problems are more likely to be present when the impacts of diabetes are more severe. There was also evidence that the instruments measure overlapping constructs relevant in Type 2 diabetes to some extent, but there is still clear divergence and evidence of disagreement between the GPBMs and the DHP-18 across the severity scale. Further evidence about the responsiveness of the measures is required.

The results support the use of both the condition specific DHP-18 and EQ-5D and SF-6D in studies requiring the assessment of HRQL and psychosocial functioning in diabetes and there is evidence that using both a generic and condition specific measure will provide a more holistic assessment of the HRQL impacts of diabetes and related treatments. This is because the measures have some level of sensitivity to diabetes specific health concerns, and the results suggest some overlap in terms of the constructs measured which are of relevance to people with diabetes. However there is also clear divergence observed at the dimension level, where a range of areas of HRQL are assessed. Therefore the use of the measures alongside each other may increase the accuracy of outcomes assessment in Type 2 diabetes by enabling the measurement of generic health concerns alongside diabetes specific indicators. This is because the GPBMs may allow for a wider assessment of HRQL.

With regard to responsiveness, both the EQ-5D and SF-6D perform better in the groups who self-report health change, although all three measures had low SRMs indicating a generally low level of responsiveness. This low level of sensitivity could be problematic in the assessment of change in QALYs before and after interventions. However, this finding could be due to the study design and sample used, which was not testing a specific intervention, but was a population survey testing a change in service structure, where health may not be expected to change for all respondents between baseline and follow up. Secondly, the measure of change used was a self-report generic question which may not have a strong relationship with changes on generic or diabetes specific PROMs. It may be important to investigate responsiveness in more detail using diabetes specific indicators of health change. Recently, a five level version of EQ-5D (EQ-5D-5L) [23] has been developed, and this may increase the sensitivity of the instrument to change over time. However direct utility values for EQ-5D-5L are not yet available.

Another key finding of this work is the strong relationship between the EQ-5D and SF-6D which has been found for diabetes [11] but is not consistently found across other health conditions [24]. The utility values derived from the measures were similar, but due to differences in the range of the utility scale (where SF-6D has a much smaller range) the spread of values differed. This affects agreement at the more severe end of the utility scale, where less SF-6D values are available, and this has been found elsewhere using similar methods [25]. The utility scales were well correlated and at the dimension level, the correlations across similar dimensions indicates overlap in the constructs being measured. Both GPBMs also displayed evidence of distinguishing between clinical and severity groups. This means that both measures have a level of validity for use in Type 2 diabetes, and the values from both instruments could be used in the estimation of QALYs with some confidence. The overlap between the measures means that there is not the requirement to include both in surveys, and there are advantages and disadvantages to both. EQ-5D is short and easy to complete, and is accepted by NICE for use in the economic evaluation of interventions. The SF-6D is derived from the SF-36 or SF-12, and therefore requires this to be included, but these measures also provide detailed information about the HRQL of patient samples.

There are a number of limitations to this study which should be considered when interpreting the findings. Firstly, psychometric validity is difficult to prove as there is no gold standard for the measurement of outcomes against which to compare the measures. Therefore validity can only be inferred against other indicators and across the instruments. Secondly, the findings are limited to the sample used which has specific characteristics which may impact on findings, particularly in relation to the level of responsiveness that should be expected in a population survey. Further work should be done to test the validity and responsiveness of EQ-5D, SF-6D and DHP-18 in relation to other diabetes specific PROMS and clinical indicators using a range of patient samples (including clinical trials to assess responsiveness in more detail). This strategy has been used in the assessment of the EQ-5D and SF-6D across mental health conditions [26]. Psychometric evidence is one method of assessing validity, and should be considered alongside other evidence to build up a picture of the measures performance. This study complements an earlier systematic review that found support for the construct validity of EQ-5D [10]. Qualitative work could also be used to assess whether all of the HRQL issues of importance to people with diabetes are assessed by the PROMS that are used for the condition (see, for example Brazier et al. [27] who used this approach in mental health. Finally, the results are limited to Type 2 diabetes, and further assessment of the GPBMs and Type 1 diabetes specific PROMs is warranted.

## Conclusion

The psychometric assessment of the relationship between the EQ-5D, SF-6D and DHP-18 shows that all have a level of validity for use in Type 2 diabetes, and suggests that the measures can be used alongside each other to provide a more holistic assessment of the HRQL issues that are important to people with Type 2 diabetes. We recommend that both generic and condition specific measures are used to assess health status in diabetes.

## Abbreviations

HRQL: Health-related quality of life; PROMS: Patient reported outcome measures; EQ-5D: EuroQol-5 dimension; SF-6D: Short form-6 dimension; DHP-18: Diabetes health profile - 18; QALY: Quality adjusted life year; VAS: Visual analogue scale.

## Competing interests

Keith Meadows developed the DHP-1 and the DHP-18, and is the founder and director of DHP Research and Consultancy Ltd. Brendan Mulhern is a member of the EuroQol Group.

## Authors’ contributions

BM and KM were involved in all stages of the analysis and interpretation of the data and were responsible for drafting and editing the submitted manuscript. Both authors read and approved the final manuscript.
